# Identification of the Immune Cell Infiltration Landscape in Head and Neck Squamous Cell Carcinoma (HNSC) for the Exploration of Immunotherapy and Prognosis

**DOI:** 10.1155/2022/6880760

**Published:** 2022-12-28

**Authors:** Chunli Huang, Jifeng Liu

**Affiliations:** ^1^Operating Room, West China Hospital, West China School of Nursing, Sichuan University, Chengdu, China; ^2^Department of Otolaryngology Head and Neck Surgery, West China Hospital, Sichuan University, Chengdu, China

## Abstract

It is generally believed that the majority of head and neck cancers develop in the mucosal epithelial cells of the mouth, pharynx, and larynx, which is collectively known as head and neck squamous cell carcinoma (HNSC). As a complex pathological process, HNSC develops through a variety of cellular and molecular events. Cancerous cells and immune cells infiltrating tumors are the main components of the tumor microenvironment. However, infiltration of HNSCs by the immune system has not been determined to date. In this work, we proposed computational algorithms to identify different immune subtypes. An analysis of the Cancer Genome Atlas (TCGA) database revealed gene expression profiles and corresponding clinical information. In HNSC patients, two immune-related genes (ZAP70 and IGKV2D-40) may be targets for immunotherapy, and these genes appear to be closely related to the prognosis. Several immunological subtypes were associated with immune function, immune checkpoints, and prognostic factors in HNSCs. Furthermore, ZAP70 is closely related to the overall survival (OS), progress-free interval (PFI), and disease-specific survival (DSS) of HNSC patients. The potential pathways that are associated with ZAP70 were found to have included adaptive immune response, response to oxidative stress, DNA replication, and lipid binding. This study provides a theoretical foundation for developing immunotherapy drugs for HNSC patients. By evaluating larger cohorts, we can gain a deeper understanding of immunotherapy and provide direction for current research on immunotherapy strategies in HNSCs.

## 1. Introduction

In the upper aerodigestive tract, head and neck cancer includes malignancies of the oral cavity, pharynx, and larynx, as well as malignancies of the paranasal sinuses and nasal cavity, and salivary glands [1]. Head and neck cancer ranks sixth in terms of new cancer cases and deaths, accounting for 2.5% of new cancer cases and 1.9% of cancer deaths worldwide [2]. According to the American Cancer Society, 64,690 new cases of head and neck cancer were diagnosed in 2017 and 13,740 of those deaths were due to the disease [3]. More than 90 percent of head and neck cancers are squamous cell carcinomas, which occur in squamous cells on the mucosal surface [4]. Head and neck cancer is most commonly caused by smoking and alcohol consumption. Several epidemiological studies have shown that high-risk human papillomaviruses play an important role in the development of certain types of head and neck squamous cell carcinomas [5]. It is believed that on-site carcinogenesis and genomic complexity drive therapeutic drug resistance in these types of cancers [6]. A combination of radiochemotherapy and high-dose cisplatin is the gold standard for treating locally advanced head and neck squamous cell carcinoma without surgery [7]. The majority of locally advanced head and neck cancers recur despite the comprehensive treatment of surgery, radiotherapy, and chemotherapy [8]. Consequently, patients with recurrent and/or metastatic head and neck squamous cell carcinoma have a poor prognosis.

Previously, different combinations of cytotoxic chemotherapy drugs have not proved to prolong the overall survival (OS) of these patients. There are three types of immune checkpoint inhibitor therapy (ICIs): programmed death ligand 1 (PD-L1), programmed death 1 (PD1), and CTL-associated protein 4 (CTLA4) [9]. The use of anti-PD1 therapy in patients with head and neck squamous cell carcinoma (HNSC) has proven to be a promising treatment for patients who have recurrences/metastases [10]. There is, however, a major limitation to ICI treatment is the low response rate among HNSC patients. A number of factors, including the immune microenvironment (TME), can influence the effectiveness of ICI, and few biomarkers can predict a patient's prognosis [11]. In HNSC patients, identifying prognostic markers associated with therapeutic benefits may allow for the individualization of immunotherapy [12]. Sadly, we know very little about the TME of HNSC, and we need better prognostic and therapeutic indicators as soon as possible.

In recent years, the bioinformatics analysis method has been applied in disciplines. For HNSC patients, an immune-related prognosis model may be useful in identifying the prognosis, molecular characteristics, and immune benefits of ICI treatment in HNSCs [13]. In addition, biomarkers, such as genetic characteristics, PD-L1 expression, PD-L2 expression, and interferon *γ*, can predict the effectiveness of checkpoint inhibitor therapy [14]. Patients can be stratified based on their prognosis and selected according to their likelihood to benefit from these treatments based on relevant prognostic models [15]. However, the research works based on the relationship between immunotherapy and HNSC are still limited. Therefore, it is urgent to discover promising biomarkers to provide better immunotherapy effects for HNSC patients. In this work, in order to explore the potential biomarkers for better immunotherapy for HNSC patients, we obtained the mRNA expression data and the clinical information of HNSC patients from TCGA database. In addition, by constructing the prognostic prediction model, we finally explore the genes that are closely associated with HNSC patients. Finally, we also explore the pathways that are highly correlated with the key genes to further reveal the relevant mechanism.

## 2. Methods

### 2.1. Dataset Downloaded

The Cancer Genome Atlas (TCGA) was launched in 2006 by the National Cancer Institute. It is an important resource for cancer researchers because it contains clinical data, genomic variation, mRNA expression, miRNA expression, methylation, and other data on different types of cancer. In this work, the expression data and clinical characteristics of HNSC in TCGA (TCGA-HNSC) were downloaded. We finally obtained a total of 502 expressions of HNSC patients and 44 expression data of normal people.

### 2.2. Differential Expression Genes in TCGA-HNSC Cohort

A dataset of RNAseq data and clinical information for HNSC was retrieved from TCGA database (https://portal.gdc.com). For studying differential mRNA expression, we used the Limma software package of *R*. An adjusted *P* value was calculated in TCGA to correct false positives. “Adjusted *P* < 0.05 and log2 FC > 1 or log2 FC < −1” was defined as the screening of threshold mRNA differential expression.

### 2.3. Enrichment Pathway Analysis Based on Gene Ontology (GO) and Kyoto Encyclopedia of Genes and Genomes (KEGG)

We investigated the most relative pathways between HNSCs based on our analysis of key genes. Furthermore, molecular function (MF) and the biological process (BP) were included in the GO enrichment analysis along with cellular components (CC). In addition, KEGG enrichment was also applied in this work. *P* < 0.05 was considered statistically significant for pathways.

### 2.4. Classification of Different Immune Subtypes

In order to quantify the individual score of each tumor case, we conducted a single sample gene set enrichment analysis (ssGSEA). The overexpression metric of the list of genes of interest compared to other genes in the genome is calculated by ssGSEA based on a rank-based method. Microarray data or log-transformed RNA sequences were used to calculate the ssGSEA score. The next step is to classify HNSCs according to immune biological feature enrichment levels (ssGSEA score) and check their tumor purity and immune score.

### 2.5. Immune Cell Infiltration Analysis

Through the use of the CIBERSORT algorithm, 22 immune infiltrating cells have been determined in different subgroups of HNSC patients. The relationship between gene expression and immune cell infiltration was then determined using Spearman correlation analysis. *P* values of 0.05 or less were considered statistically significant.

### 2.6. Gene Set Variation Analysis (GSVA)

GSVA, a nonparametric, unsupervised method, was used to evaluate gene set enrichment. By scoring the gene set of interest, we transformed gene-level changes into pathway-level ones. Next, we determined the sample's biological function. In the present study, the gene sets were retrieved from the molecular signature database. With GSVA, we conducted a comprehensive assessment of potential changes in biological functions in various samples.

### 2.7. Drug Sensitivity Analysis

With the “pRRophetic” *R* package, the chemotherapy sensitivity of each tumor sample was predicted using the Genomics of Drug Sensitivity in Cancer database (GDSC). Further analysis of each chemotherapy drug's IC50 values was conducted using regression analysis. Cross-validation was performed ten times on the GDSC training set to test the regression and prediction accuracy. The parameters were all set to their default values, including the “combat” parameter, which averages repeated gene expressions to remove batch effects.

### 2.8. Statistical Analysis

The Cox proportional hazard model was used to analyze multivariate data. The log-rank test was used to compare survival curves calculated using Kaplan–Meier methods. All statistical analyses were performed using *R* software. Statistical significance was determined by *P* values less than 0.05 on both sides.

## 3. Results

### 3.1. A Large Number of Differentially Expressed Genes Were Explored between the HNSC Cohort and the Normal Cohort

**Figure 1:**
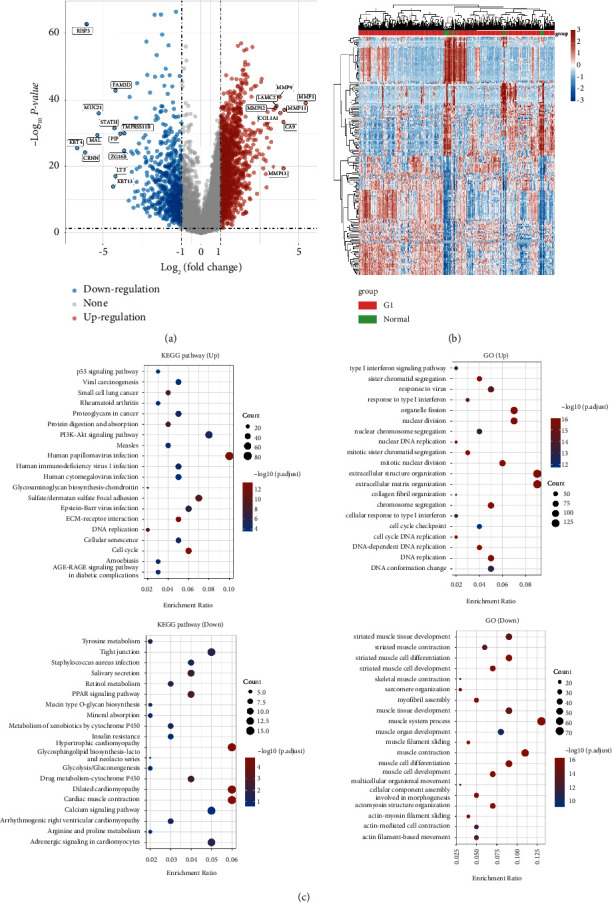
(a) The differentially expressed analysis between the HNSC cohort and normal people; (b) the heatmap demonstrated the differentially expressed genes; (c) the GO and KEGG enrichment analysis based on the differentially expressed genes.

Firstly, the mRNA expression data of the HNSC cohort and normal cohort were downloaded from TCGA database. Subsequently, after data processing, deduplication and merging into the matrix, we obtained a matrix with 502 HNSC patients and 44 normal people. Then, we performed the differentially expressed analysis between two different cohorts. The results demonstrated that 2234 genes were considered differentially expressed genes, which included 1624 up-regulated genes and 610 down-regulated genes (Figures 1(a)-1(b)). We also evaluated the GO and KEGG enrichment analysis based on these differentially expressed genes. The KEGG enrichment analysis revealed that p53 signaling pathways, viral carcinogenesis, proteoglycans in cancer, and the PI3K-AKT signaling pathway were positively associated with up-regulated differentially expressed genes, while tyrosine metabolism, tight junction, retinol metabolism, the PPAR signaling pathway, and drug metabolism were highly correlated with down-regulated differentially expressed genes. For GO enrichment analysis, the type I interferon signaling pathway, response to virus, nuclear DNA replication, and response to type I interferon were closely associated with up-regulated differentially expressed genes. However, striated muscle tissue development, muscle system process, muscle cell differentiation, and muscle cell development were correlated with down-regulated differentially expressed genes (Figure 1(c)).

### 3.2. Different Immune Subtype Analyses Showed the Two Immune Groups in TCGA-HNSC Cohort

**Figure 2:**
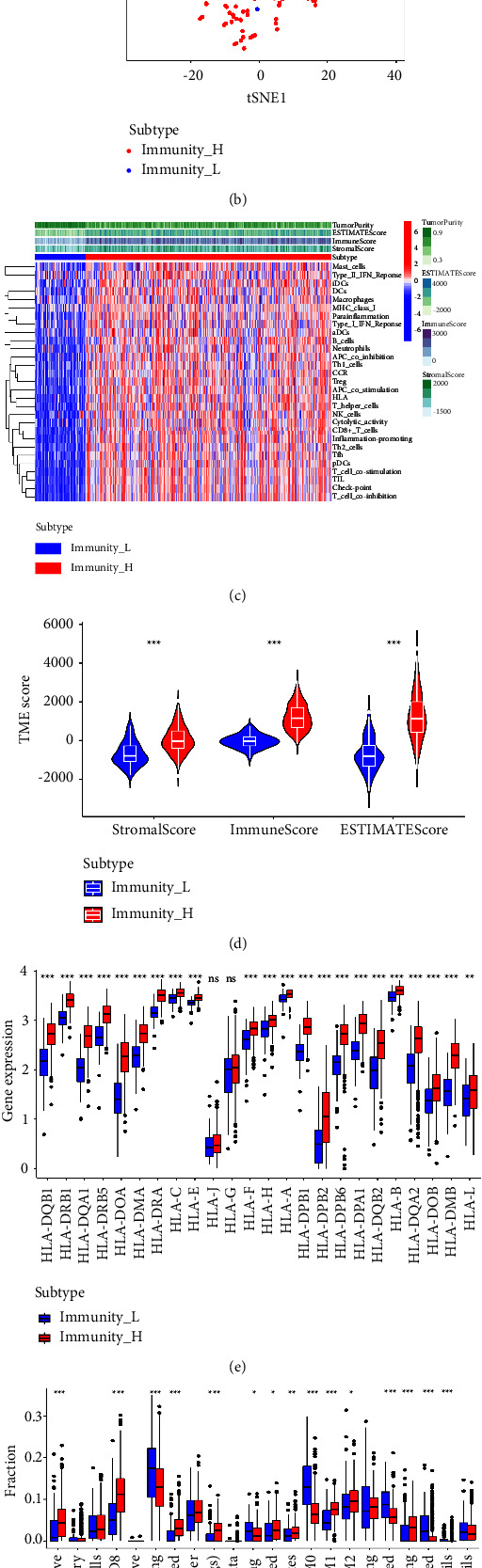
(a) The immune subtype analysis based on the different expression level of immune-related genes; (b) the t-SNE's dimensionality reduction algorithm showed that the subtypes were highly consistent with the two-dimensional distribution pattern; (c) the heatmap reveals different immune scores, immune-related cell distribution, and tumor cells between Immunity_L and Immunity_H groups; (d) the stromal score, immune score, and estimate score between Immunity_L and Immunity_H groups; (e) the expression level of HLA-related genes between Immunity_L and Immunity_H groups; (f) the different immune cells infiltration between Immunity_L and Immunity_H groups.

Subsequently, in order to construct the different immune subtypes for the better exploration of immunotherapy of HNSC patients in TCGA cohort, TCGA datasets containing HNSC samples were clustered using the ssGSEA method based on immune cells (Figure 2(a)). At a *k*-value of two, the t-SNE's dimensionality reduction algorithm showed that the subtypes were highly consistent with the two-dimensional distribution pattern (Figure 2(b)). In the Immunity_L group, low immunity was characterized by low immunity, and in the Immunity_H group, high immunity was characterized by high immunity. In the Immunity_L group, the heatmap demonstrated that more tumor-associated cells were enriched and fewer immune-related cells were enriched. However, the less tumor-associated cells and more immune-related cells were shown in the Immunity_H group (Figure 2(c)). Additionally, we also evaluate the tumor microenvironment score in Immunity_L and Immunity_H groups. The results revealed that the Immunity_H group is associated with a higher stromal score, immune score, and estimate score compared with the Immunity_L group (Figure 2(d)). A total of 24 genes that encode human leukocyte antigens (HLA) were examined in our study. Immune HLA gene expression was significantly lower in the Immunity_L group, suggesting that tumor cells present antigenicity in a compromised manner in order to evade immune surveillance. We then performed the immune cell infiltration analysis based on the CIBERSORT algorithm. Compared with the Immunity_L group, more naive B cells, CD8 T cells, and regulatory T cells were shown in the Immunity_H group. In addition, more CD4 memory T cells, M0 macrophages, and activated dendritic cells are shown in the Immunity_L group (Figures 2(e)-2(f)).

### 3.3. Construction of the Prognostic Prediction Model Based on the HNSC Cohort

**Figure 3:**
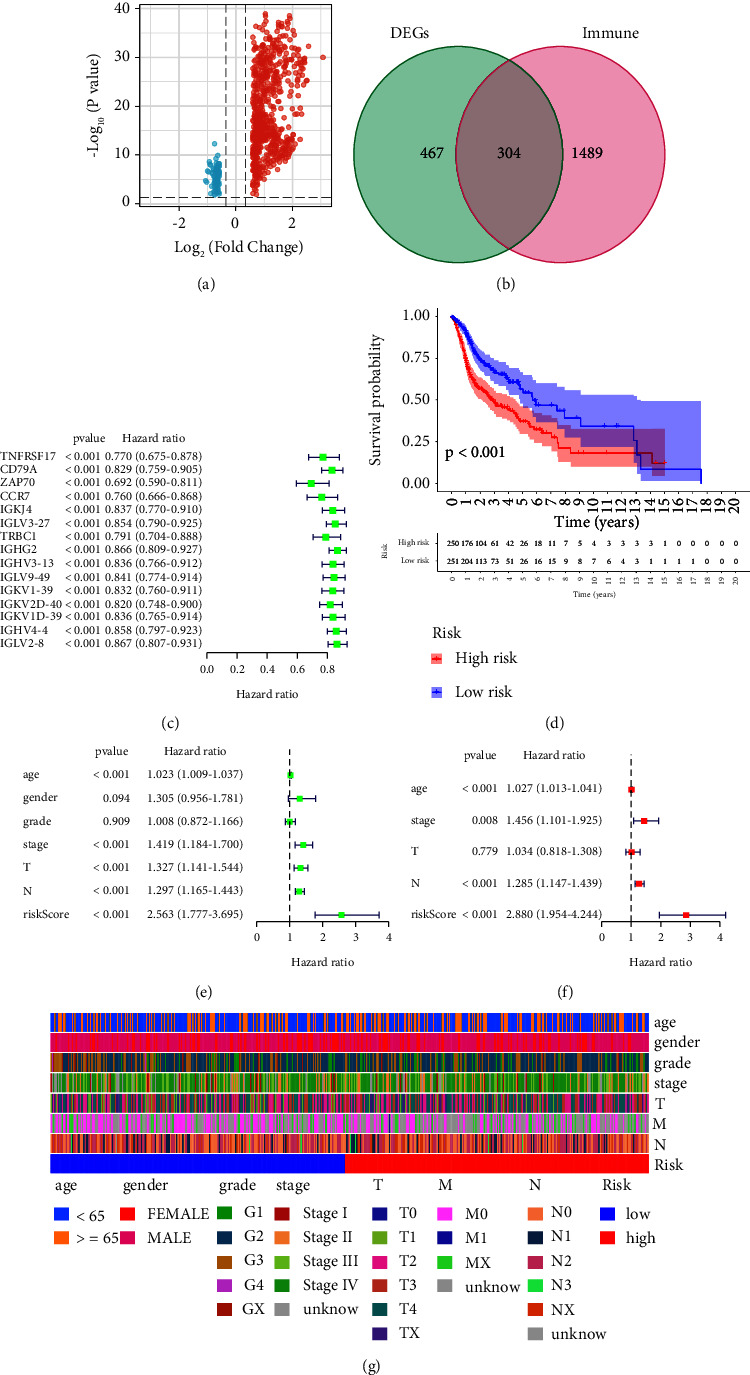
(a) The volcano map was used to evaluate the differentially expressed genes between Immunity_L and Immunity_H groups; (b) the Venn diagram was applied to obtain the genes that are involved in differentially expressed genes and immune-related genes; (c) the univariate COX regression analysis was based on the key genes; (d) the survival analysis between low-risk and high-risk groups; (e) the univariate independent prognostic analysis based on clinical characteristics and risk scores; (f) the univariate independent prognostic analysis based on clinical characteristics and risk scores; (g) the heatmap reveals the relationship between the risk score and clinical characteristics.

**Figure 4:**
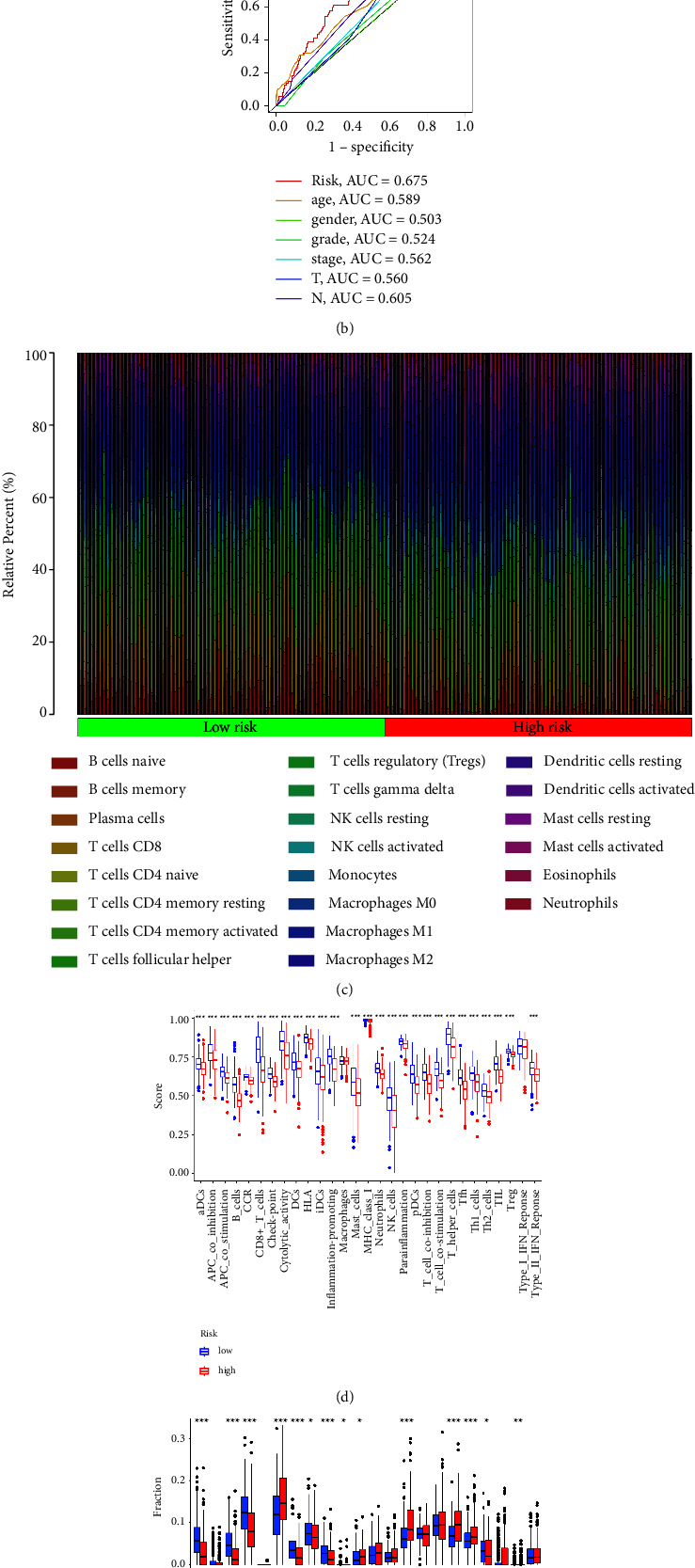
(a) The time-dependent ROC curves reveal the prognostic prediction value of the model; (b) the ROC curve demonstrated the prognostic prediction value of risk scores and clinical characteristics; (c) the different immune cell infiltration between low-risk and high-risk groups; (d) different immune-related functions between low-risk and high-risk groups; (e) the boxplot reveals the correlation between low-risk and high-risk groups.

Firstly, on the basis of Immunity_L and Immunity_H groups, we performed the differentially expressed analysis. We finally obtain a total of 771 differentially expressed genes, which includes 82 down-regulated genes and 689 up-regulated genes (Figure 3(a)). Subsequently, we evaluate the expression level of 1793 immune-related genes. The Venn diagram revealed that 304 of them were considered differentially expressed genes (Figure 3(b)). After combining the expression data and the clinical characteristics of HNSC patients, we then constructed a prognostic prediction model. The univariate COX regression analysis revealed that a total of 15 immune-related genes were proved to be closely associated with the prognosis of HNSC patients, including TNFRSF17, CD79A, ZAP70, CCR7, IGKJ4, IGLV3-27, TRBC1, IGHG2, IGHV3-13, IGLV9-49, IGKV1-39, IGKV2D-40, IGKV1D-39, IGHV4-4, and IGLV2-8 (Figure 3(c)). Subsequently, we then performed the multivariate COX regression analysis. The results demonstrated that two genes (ZAP70 and IGKV2D-40) were considered to be highly correlated with the prognosis of HNSC patients. The risk score = ZAP70 *∗* −0.258460790509476 + IGKV2D-40 *∗* −0.128579397384237. On the basis of the risk score, the patients were divided into low-risk and high-risk groups. The survival analysis showed that HNSC patients involved in the low-risk group are associated with better OS compared with HNSC patients with a lower risk score (Figure 3(d)). In addition, we also evaluated the prognostic value of the prognostic prediction model. The univariate independent prognostic analysis revealed that age, stage, T stage, N stage, and the risk score are the independent risk factors for HNSC patients. For multivariate independent prognostic analysis, the age, stage, N stage, and the risk score are the independent prognostic factors for HNSC patients (Figures 3(e)–3(g)). Subsequently, we evaluate the relationship between clinical factors and risk scores. The ROC curve demonstrated that the AUC score for 1-year, 3-year, and 5-year for HNSC patients is 0.675, 0.641, and 0.642, respectively. In addition, the clinical ROC curves demonstrated that risk scores showed better predictive values than age, gender, grade, stage, N stage, and T stage in the HNSC cohort (Figures 4(a)-4(b)).

### 3.4. Immune Cell Infiltration and Immune Functions between Low-Risk and High-Risk Groups

**Figure 5:**
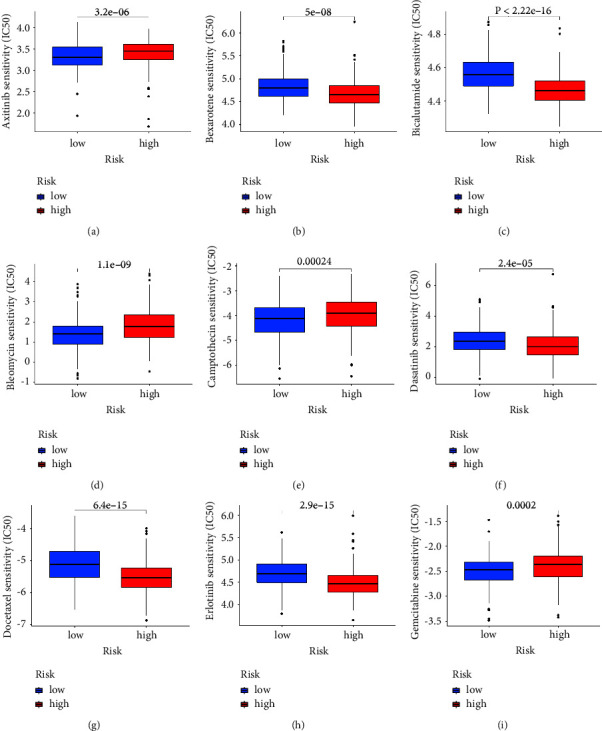
The drug sensitivity analysis of (a) axitinib, (b) bexarotene, (c) bicalutamide, (d) bleomycin, (e) camptothecin, (f) dasatinib, (g) docetaxel, (h) erlotinib, and (i) gemcitabine.

Then, we evaluate the correlation between the risk score and immune functions. The results demonstrated that most immune-related functions are significantly different between low-risk and high-risk groups, including immune checkpoints, HLA-related genes, type I IFN response, type II IFN response, cytolytic activity, APC coinhibition, and APC costimulation. For immune-related cells, the results revealed B naive cells, plasma cells, CD8 T cells, CD4 memory T cells, regulatory T cells, NK cells, macrophages, dendritic cells, and mast cells (Figure 4(c)–4(e)). In addition, we also evaluate the relationship between the risk score and drug sensitivity. The results suggested that the risk score is associated with a large number of drug sensitivity, including axitinib, bexarotene, bicalutamide, bleomycin, camptothecin, dasatinib, docetaxel, erlotinib, and gemcitabine. The drug sensitivity analysis revealed that the risk score could be regarded as a good predictive factor for the treatment of chemistry medicine (Figure 5).

### 3.5. Exploration of the Role of ZAP70 in the HNSC Cohort

**Figure 6:**
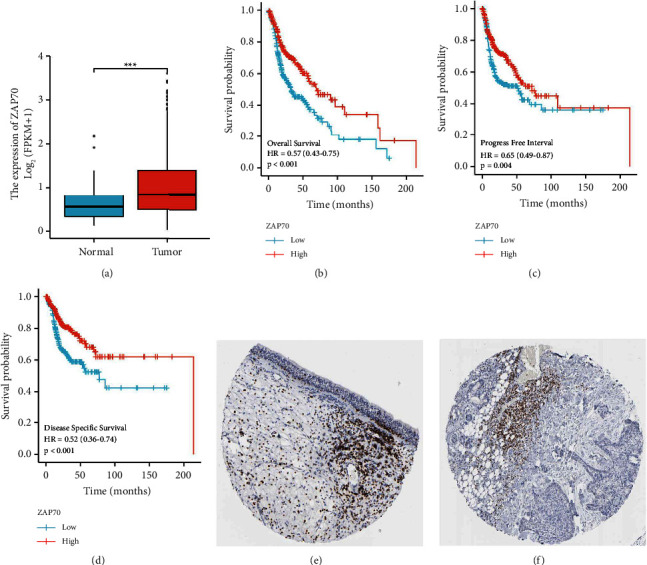
(a) The expression level of ZAP70 in normal and tumor tissues; (b) the OS between low-risk and high-risk groups; (c) the PFI between low-risk and high-risk groups; (d) the DSS between low-risk and high-risk groups; (e) the immunohistochemical results of ZAP70 in normal tissues; (f) the immunohistochemical results of ZAP70 in tumor tissues.

**Figure 7:**
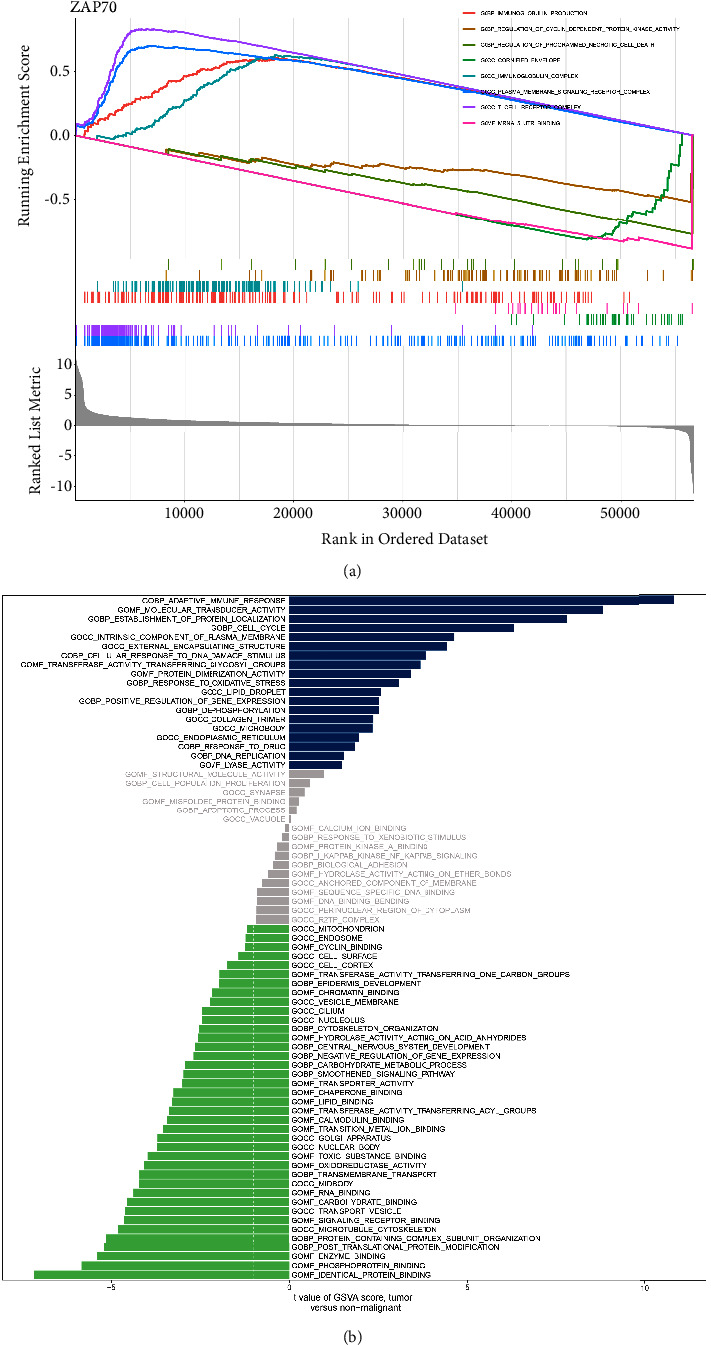
(a) The GSEA results of ZAP70 in TCGA-HNSC cohort; (b) the GSVA results between low and high expression of ZAP70 groups.

Based on the previous analysis, we discovered that ZAP70 may play an important role in HNSC patients. The differentially expressed analysis revealed that ZAP70 is significantly up-regulated in the tumor group compared with the normal group (Figure 6(a)). The survival analysis demonstrated that the high expression level of ZAP70 is closely associated with poor OS, progress-free interval (PFI), and disease-specific survival (DSS) (Figures 6(b)–6(d)). The immunohistochemical results suggest that there is no significant difference between HNSC tissue and normal tissue (Figures 6(e)-6(f)). For GSEA, the results suggested immunoglobulin production, regulation of cyclin-dependent protein kinase activity, regulation of programmed necrotic cell death, cornified envelope, immunoglobulin complex, and T-cell receptor complex. The results of GSVA revealed that many pathways are closely associated with high expression levels of ZAP70, including adaptive immune response, molecular transducer activity, cell cycle, response to oxidative stress, DNA replication, and the microbody. In addition, the low expression level of ZAP70 is closely associated with cyclin binding, endosome, lipid binding, toxic substance binding, and RNA binding (Figures 7(a)-7(b)).

## 4. Discussion

A malignant tumor of the upper respiratory tract and digestive tract, including the oral cavity, the nasopharynx, the oropharynx, the hypopharynx, and the larynx, is known as HNCS. Most HNCS cases are caused by squamous cell carcinomas (SCC) [16]. Smoking and chewing tobacco are considered to be important pathogenic factors for HNCS development. Despite progress in treatment methods, such as surgery, chemotherapy, and radiotherapy, the 5-year survival rate has not improved significantly [17]. For these reasons, new biomarkers are urgently needed for effective diagnosis and prognosis evaluation, as well as for developing effective treatment strategies. In this work, we aim to explore the genes that could be considered potential immunotherapy targets for HNSC patients by bioinformatics analysis.

Genome changes play a significant role in cancer etiology. Alterations can manifest as abnormal insertions, deletions, or substitutions of nucleotides or chromosomes, resulting in abnormal phenotypes. As a result, genomic biomarkers are valuable for predicting changes in tumor biology during and after chemotherapy and can also be used as therapeutic targets [18]. Firstly, by performing the differentially expressed analysis, we discovered that a number of genes were regarded as differentially expressed genes. The GO and KEGG enrichment analysis also revealed that many potential pathways are closely associated with HNSC. Subsequently, based on the immune-related genes, the HNSC cohort was divided into Immunity_L and Immunity_H groups. The immune-related cells and immune-related function analysis demonstrated that a significant difference was discovered between Immunity_L and Immunity_H groups. As cancer immunogenomics and immunotherapy have developed over the past decade, tremendous progress has been improved. The combination of checkpoint blockade and its established efficacy and safety profile with more novel immunomodulatory drugs makes up an important component of current HNSC clinical trials [19]. There are a number of monoclonal antibodies being developed that target immune-suppressive pathways other than PD-1 and CTLA-4. A LAG-3 inhibitor, relatlimab, has recently entered clinical trials for treating HNSC with or without nivolumab, showing promising preclinical results [20].

In order to explore the potential targets for the better treatment of HNSC patients, we then construct the prognostic prediction model. Based on the univariate COX regression and multivariate COX regression analysis, we finally obtain the genes that are closely associated with the prognosis of HNSC patients, which includes ZAP70 and IGKV2D-40. In recent years, many studies focused on bioinformatics analysis to explore the genes that play an important role in the prognosis of tumor patients [21–25]. There was a correlation between nivolumab response and HNCAF-0/3 of fibroblast subtypes, whereas HNCAF-1 caused immunosuppression [26]. Based on predictive computational models that include PD-L1 and immunosuppressive biomarkers, Bates et al. propose how HNSCC patients can be stratified according to how likely they are to respond to immunotherapy [27].

Subsequently, we further explore the role of ZAP70 in the HNSC cohort. The differentially expressed analysis revealed that ZAP70 is significantly up-regulated in the HNSC patients compared with normal people. The survival analysis also suggested that the high expression level of ZAP70 is closely associated with poorer OS, DSS, and PFI. However, the immunohistochemical suggests that the expression level of ZAP70 encoded protein shows no difference between the HNSC cohort and normal people. The function analysis revealed that many enriched pathways are closely associated with ZAP70, such as adaptive immune response, response to oxidative stress, DNA replication, and lipid binding. High levels of tumor immune infiltration were observed in HNSCs. A high density of tumor-infiltrating lymphocytes is associated with improved outcomes in HNSCs. It has been demonstrated in previous studies that high densities of CD3, CD8, and CD57 cells in the immune infiltrate are associated with an improved OS and PFS after immunotherapy [28]. NK cells that are particularly effective against HNSCs are characterized by CD3 and CD8, which are selective markers for T-lymphocytes and cytotoxic T-lymphocytes. Recently, several studies have found that oxidative stress plays an important role in the diagnosis and treatment of HNSC patients [29]. Inhibition of NOX2 by HPV16 E6 and E7 proteins leads to genomic instability, increased DNA damage susceptibility, and genomic instability in head and neck cancer cells [30]. Additionally, another study has discovered that human head and neck cancer cells exposed to 2DG in combination with cisplatin exhibit enhanced cytotoxicity [31].

In conclusion, based on the different immune subtypes, we obtained provided new directions for immunotherapy for HNSC patients. In addition, the prognostic prediction model demonstrated that ZAP70 and IGKV2D-40 may be closely associated with the prognosis of HNSC patients. Furthermore, ZAP70 is closely related to the OS, DSS, and PFI of HNSC patients. The potential pathways that are associated with ZAP70 were found to have included adaptive immune response, response to oxidative stress, DNA replication, and lipid binding. Our research provided a new target for immunotherapy for HNSC patients [13].

## Data Availability

The data used to support the findings of this study could obtain from the corresponding author.
